# Parallel evolution of picobirnaviruses from distinct ancestral origins

**DOI:** 10.1128/spectrum.02693-23

**Published:** 2023-10-27

**Authors:** Lester J. Perez, Gavin A. Cloherty, Michael G. Berg

**Affiliations:** 1 Infectious Disease Core Research, Abbott Diagnostics Division, Abbott Laboratories, Abbott Park, Illinois, USA; 2 Abbott Pandemic Defense Coalition (APDC), Chicago, Illinois, USA; US Food and Drug Administration, Silver Spring, Maryland, USA

**Keywords:** Picobirnavirus, convergent evolution, functional divergence, adaptive divergence, RdRp, capsid

## Abstract

Picobirnaviruses (PBVs) are small, bi-segmented, double-stranded RNA viruses frequently associated with gastrointestinal and recently linked to respiratory infections. Detected in hosts from distant biological kingdoms, debate swirls as to their age and origins and whether they are prokaryotic or eukaryotic viruses. Our evolutionary analysis revealed a contemporaneous emergence for PBV, as PBV-R_1&3_ species arose ~350 years ago with both segments, whereas the more ancient species, PBV-R_2_, initially lacked capsid. Integrated phylogenetic reconstruction defined two origins for PBV, determining PBV-R_1&3_ species descended from *Reovirus* and PBV-R_2_ branched with *Partitivirus* ancestors. These results, coupled with the heterogeneity of Shine-Dalgarno motifs, argue against a prokaryotic origin. Epistatic interactions identified in the RdRp of PBV-R_1&3_ evidenced the constraints imposed by vertebrate host immunity, whereas its absence in PBV-R_2_ concurs with its fungal origin. After acquisition of capsid, PBV-R_2_ increased its adaptive and functional divergences in RdRp domains and the compactness of its RNA structure to enable encapsidation. While their pathogenicity remains an open question, picobirnaviruses likely originated from both fungal and avian hosts: parallel evolution mechanisms have driven the genetic similarities shared among present-day PBV species.

**IMPORTANCE:**

Picobirnaviruses (PBVs) are highly heterogeneous viruses encoding a capsid and RdRp. Detected in a wide variety of animals with and without disease, their association with gastrointestinal and respiratory infections, and consequently their public health importance, has rightly been questioned. Determining the “true” host of Picobirnavirus lies at the center of this debate, as evidence exists for them having both vertebrate and prokaryotic origins. Using integrated and time-stamped phylogenetic approaches, we show they are contemporaneous viruses descending from two different ancestors: avian *Reovirus* and fungal *Partitivirus*. The fungal PBV-R2 species emerged with a single segment (RdRp) until it acquired a capsid from vertebrate PBV-R1 and PBV-R3 species. Protein and RNA folding analyses revealed how the former came to resemble the latter over time. Thus, parallel evolution from disparate hosts has driven the adaptation and genetic diversification of the *Picobirnaviridae* family.

## INTRODUCTION

Picobirnaviruses (PBVs) are double-stranded RNA (dsRNA) viruses found in a variety of vertebrate species (1). They are believed to be causes of diarrhea and gastroenteritis and are frequently observed in post-transplantation and immunocompromised individuals (2). Recently, we and others have detected PBV in nasal secretions and in patients hospitalized with respiratory disease (3, 4). A major open question is whether PBV is a vertebrate virus or simply a non-chordate eukaryote (fungal, parasitic) or prokaryotic (bacterial, fungal) virus that is coincident with these primary infections (5, 6). The presence of Shine-Dalgarno (bacterial ribosome binding) sequences in the 5′UTRs of capsid and RdRp genes, its relatedness to fungal partitiviruses, and the lack of phylogenetic delineation along host lines argue in favor of the latter (7 – 9). However, PBV particles are capable of disrupting biological membranes *in vitro*, suggesting the capsid can invade animal cells (10). Indeed, its capsid undergoes autocatalytic maturation, a process observed in other non-enveloped animal viruses that activates the virion for entry. Likewise, the capsid projecting (P) domain exhibits low conservation and is exposed, likely subject to antigenic drift as it avoids adaptive immunity or seeks to utilize cellular receptors of different host species (10). A better understanding of the mechanisms driving the emergence and evolutionary processes guiding PBV diversification could shed light on this debate.

## RESULTS

### The contemporaneous emergence of PBV species is distinguished by their genomic segment composition

A maximum clade credibility (MCC) tree was constructed for RdRp and capsid to determine the evolutionary history of *Picobirnavirus*. An initial Bayesian evaluation of temporal signal (BETS) evaluation of the temporal signal yielded strong support for heterochronous over isochronous models for both genomic segments using either a strict or an uncorrelated relaxed log normal clock model with constant growth as prior (Table 1). Log marginal likelihoods calculated using both the path sampling (PS) and stepping-stone (SS) sampling methods with a Bayes factor >3 favored the heterochronous data set (Table 1). Further assessments of molecular clock models and proper priors by these methods revealed that an uncorrelated relaxed local clock model in combination with a Bayesian skyline plot prior provided the best rankings (Table S1 and S2). The topological structure of the trees and temporal distribution of strains (time-stamped distribution) together indicate these are contemporaneous viruses that emerged <700 years ago. Indeed, each tree’s ladder-like appearance (e.g., ancestral strains near the root and recent strains at distal tips) and the inferred timescale would not be expected for bacteriophages of prokaryote-linked viruses.

**TABLE 1:** BETS analysis comparing data fitting for two models: heterochronous (het) and constrained (Iso: isochronous)^*a*^

Coding region	Model	Log marginal likelihood		BF	
		PS	SS	PS	SS
*RdRp*	SChet	176,580.2855	176,567.3726		
	SCIso	176,878.692	176,899.6801	298.4064438	332.3074985
	URLChet	175,969.1107	175,969.1107		
	URLCIso	176,250.5675	176,274.2038	281.4567672	305.0930831
*Capsid*	SChet	405,357.6929	405,397.271		
	SCIso	405,449.0548	405,479.4964	91.36191103	82.22538598
	URLChet	405,085.058	405,116.8626		
	URLCIso	405,842.493	405,877.5076	757.4349536	760.6449583

^*a*^ The results are summarized from the log-marginal likelihood estimations using PS and SS algorithms.

**Fig 1:**
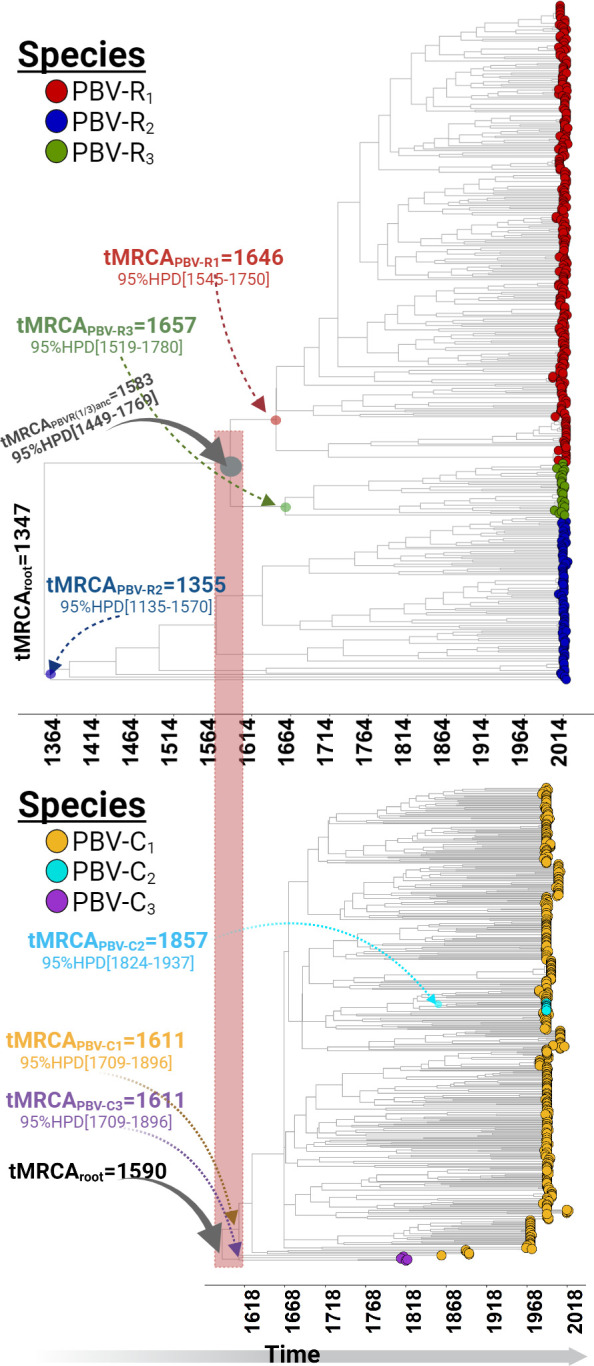
Evolutionary history of *Picobirnavirus*. Time-calibrated maximum clade credibility trees for both genome segment analysis reveal a more ancestral origin for *RdRp* (top; tMRCAroot = 1,347) compared with capsid (bottom; tMRCAroot = 1,590). Diversification into the three species defined by Perez et al. (11) is denoted with tMRCA/95%HPD interval at main internal nodes using the color scheme indicated in the caption legends. The emergence of capsid (bottom) coincided with the diversification of PBV-R_1_ and PBV-R_3_ species (top), as highlighted by the red rectangle.

Whereas the capsid phylogeny reflects a more recent, single speciation event, the RdRp revealed an older emergence, with a subsequent diversification into three co-evolving species, in agreement with the genetic analysis presented in reference 11. RdRp bifurcated immediately from the root (Fig. 1, top panel), with PBV-R_2_ (blue; tMRCA = 1,347) being the most ancestral lineage. Notably, the emergence of capsid (tMRCA = 1,590) coincided with the diversification (tMRCA = 1,583, gray node) of PBV-R_1_ (red) and PBV-R_3_ (green) species (Fig. 1, bottom panel). This immediately suggests two competing theories to explain their different evolutionary histories. In model 1 (Fig. S1A), the RdRp lineages share a common ancestor, whereupon the acquisition of capsid through a reassortment event, selective pressure drove the diversification of PBV into three species. In model 2 (Fig. S1B), the RdRp lineages descended from different ancestors, with PBV-R_1_ and PBV-R_3_ each possessing a capsid from the time of their emergence, while PBV-R_2_, initially lacking a capsid, acquired this segment later through a duplication and reassortment event with one of these two PBV species by a mechanism previously described (12).

### The PBV family is characterized by the convergent evolution of RdRp from two distinct origins

**Fig 2:**
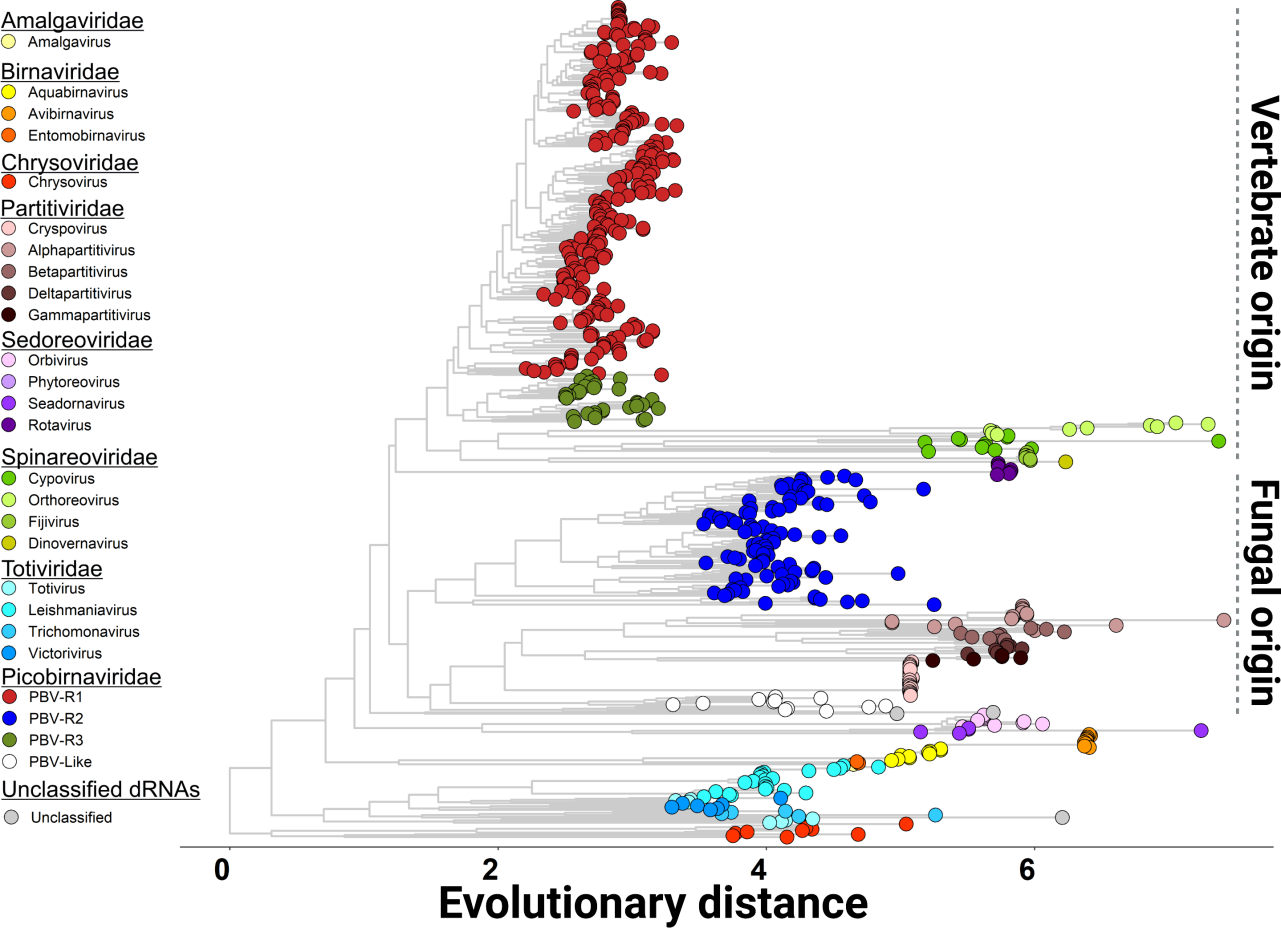
Resolved phylogenies for *Picobirnavirus* and closely related dsRNA viral families. Maximum likelihood (ML) tree reconstruction for the complete RdRp coding region using all available sequences in GenBank for PBV and closely related dsRNA viral families, as previously determined by Knox et al. (13) (see Table S7). Colors of taxa used to denote each viral family are listed (left). The phylogenetic relationship between PBV-R_1_ (red) and PBV-R_3_ (green) and *Reoviridae* suggests a vertebrate origin, whereas for PBV-R_2_ (blue), a fungal origin is proposed.

Phylogenetic trees of RdRp proteins from PBV and other segmented, dsRNA viruses were constructed to explore these hypotheses (Fig. 2). Surprisingly, PBV-R_1_ and PBV-R_3_ sequences branched with the *Spinareoviridae* family, having diversified from a common rotavirus ancestor. PBV-R_2_ grouped separately from PBV-R_1_ and PBV-R_3_ and branched with *Partitiviridae* family members that infect fungi, both (PBV-R_2_ and *Partitiviridae*) basal to “PBV-like” viruses employing an alternative, mitochondrial genetic code (9). Thus, whereas PBV-R_1_ and PBV-R_3_ appear to have a vertebrate reovirus origin, a non-chordate eukaryotic origin is inferred for the PBV-R_2_ species. In a similar previous analysis by Knox et al., PBV sequences (genogroups I nd II) remained clustered together (13). Figure 2 by comparison contains more taxa and is derived from a full-length alignment; however, the key factor altering our tree topology is the inclusion of the “PBV-like” strains absent from their analysis (13). Its removal results in all three PBV species clustering together (Fig. S2). Importantly, PBV-like viruses do not possess a capsid and they closely resemble mitoviruses which infect the mitochondria of fungi (9, 14). Thus, the evidence supports an independent evolution from different ancestral starting points converging to acquire similar traits through the diversification of each PBV species (hypothesis model 2). The data suggest PBV-R_1_ and PBV-R_3_ species containing capsids emerged more recently from a vertebrate host, whereas the more ancient PBV-R_2_ lacking a capsid acquired it from PBV-R_1_ and PBV-R_3_. These results are aligned with evidence of co-infections between PBV and reoviruses (rotaviruses) each having similar gastric pathologies, reflecting a close relationship among these viral groups (2).

### Epistatic constraints in RdRp highlight differential host-virus interactions

**Fig 3:**
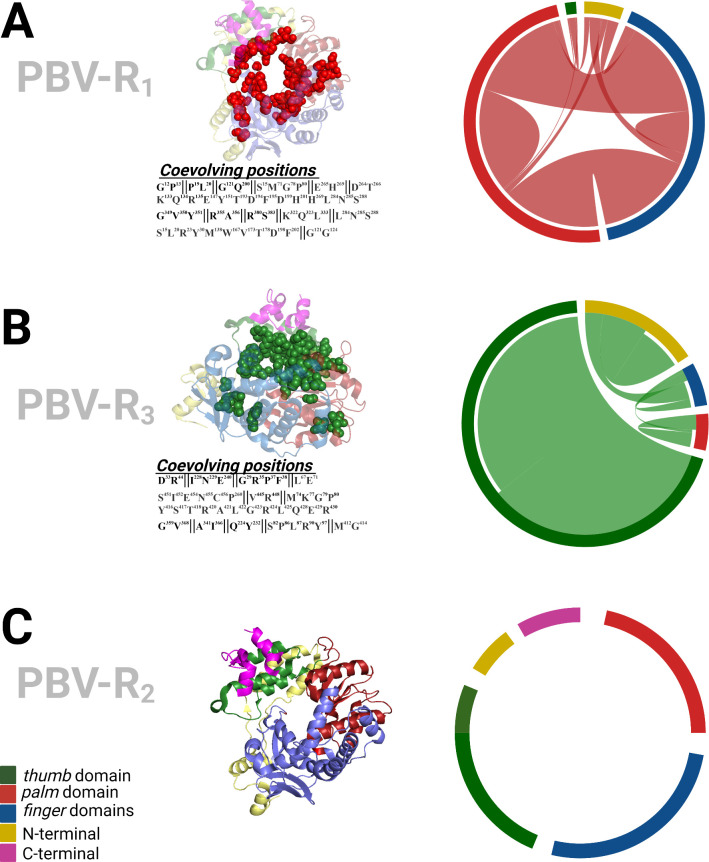
Coevolutionary events in *RdRp* of *Picobirnavirus*. BIS2 analysis was performed as described by Champeimont et al. (15) to identify amino acid pair interactions with statistical significance. For all three PBV species (A–C), coevolved residues were mapped as spheres onto the 3D structure of PBV-R1 (PBD: 5i62). Domains of the *RdRp* are colored as described in reference 16, with the N- and C-terminal domains in yellow and magenta, respectively, fingers in blue, the palm in red, and the thumb subdomain in green. Residue pairs and their sequence positions are shown beneath each 3D structure. The circular representation depicts the intra- and inter-domain interactions for each epistatic residue. The width of these links indicates the frequency of coevolutionary events as estimated by the *post hoc* summarized weight (see matrixes in Fig. S3).

A co-evolutionary analysis was performed to identify epistatic interactions maintaining PBV RdRp protein functionality and the role these constraints play in viral fitness. Clusters of statistically recognized coevolutionary pairs (Fig. S3) were mapped onto the 3D structure, and the weight of their epistatic interactions was estimated (Fig. 3). Results reflect the role of the stabilizing selection imposed on the virus by the host as an indication of the likelihood of successful adaptation. For PBV-R_1_, we observed a high degree of interconnectivity between domains, wherein residues of the palm, finger, thumb, and N-terminal domains compensate for one another when mutated to preserve functionality. PBV-R_3_ was less constrained than PBV-R_1_, displaying greater “intra”- versus “inter”-domain constraints, suggesting the host immune response against the virus has a less selective role in this process. Once again, PBV-R_2_ shows a completely different pattern: there are no intra-/inter-domain constraints and consequently no evidence of a co-evolutionary process. Therefore, based on the coevolutionary analysis, the evolution of PBV-R_1+3_ proteins were subject to epistatic modifications and appears to have been restricted by host jumping event(s) to vertebrates (avian, mammals), whereas the PBV-R_2_ protein lacks these constraints. While functional experiments will be required to corroborate the findings of the coevolutionary analysis, the results again indicate that PBV1+3 and PBV2 have different evolutionary histories.

### PBV-R_2_ lacked an extracellular virion phase during the first 300 years of its emergence

**Fig 4:**
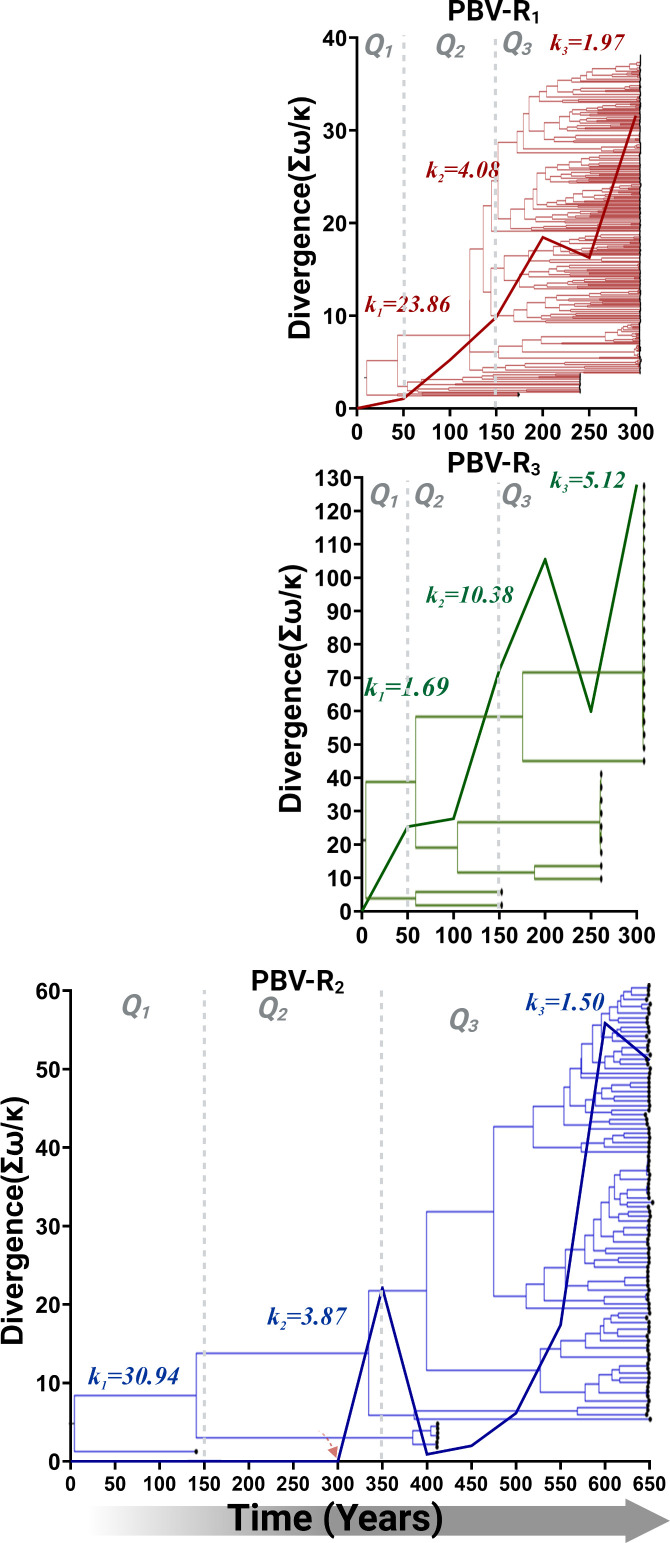
Adaptive divergence in *RdRp* of *Picobirnavirus*. Each panel contains the estimated adaptive divergence for each viral species: (A) PBV-R_1_, (**B**) PBV-R_2_, and (C) PBV-R_3_. A Maximum clade credibility (MCC) tree was obtained from a three-epoch model, with each epoch time (Q_1_, Q_2_, or Q_3_) separated by dashed gray lines. The adaptive divergence was derived by taking the sum (Σ) of positively selected branches *ω_2_ = d_N_*/*d_S_* with statistical support (when contrasting A_1_ vs A model in PAMLv9.2; Tables S4 to S6) and dividing by the kappa values (*k_n_*) (transitions/transversion values) for each epoch time in 50-year windows. The putative time for the PBV-R2 acquisition of capsid is denoted by a red arrow.

To further dissect the differences between PBV-R_1_ and PBV-R_3_ versus PBV-R_2_, we estimated species adaptive divergence over time. A time heterogenous phylogeny was obtained from a three-epoch model, wherein kappa values (*k_n_*: transitions/transversion bias) measured over 50-year windows define each epoch and provide an estimate of the accumulation of mutations during this interval (Fig. 4). Adaptive divergence (solid line) is calculated by dividing Darwinian selection (episodic selection) (*ω_2_ = d_N_*/*d_S_*) by mutational ratio bias (*k_n_* parameters). Positively selected lineages (e.g., *ω*_2_ > 1) during periods in which numerous amino acid-changing transversions accrue (e.g., low *k_n_*) are an indication of a higher probability of fixation (fixation bias) for the new lineage; hence, its adaptive divergence increases. Upon emergence 300 years ago, PBV-R_1_ kappa levels were initially elevated (*k*_1_ = 23.86). This is an indication of a high accumulation rate of mutations, potentially resulting from a host-jump event, but which steadily declines over time (*k*_2_ = 4.08, *k*_3_ = 1.97) as the virus adapts to its new presumed mammalian host (Fig. 4A). PBV-R_3_ also shows a similar trend in fixation bias and adaptive divergence (bold line) despite low mutational ratio bias early on (*k*_1_ = 1.69), a value which could be impacted by the low number of available sequences (Fig. 4C). By contrast, PBV-R_2_ shows no evidence of a positively selected lineage until late in epoch Q2, some 300 years *after* its emergence (Fig. 4B). This timeframe coincides precisely with the acquisition of the capsid segment (red arrow; see Fig. 1). Elevated kappa values (e.g., 30.94) without fixation for PBV-R_2_ in the first 150 years prior to this event reflect the unconstrained generation of mutations in RdRp in the absence of limits imposed by an RdRp-capsid interaction. These results are once again consistent with PBV-R_2_ not being under immune pressure and suggest a lifestyle without an extracellular virion phase. Either by complementarity or co-infection events, PBV-R_2_ acquired this new genomic segment and encapsidation capacity. It is still unclear if PBV-R_2_ (PBV-R_1_ and PBV-R_3_) can achieve mammalian infection on its own or only via co-infection with its natural host, but this event was pivotal in the diversification of this lineage.

### Shine-Dalgarno motifs in *Picobirnaviridae* are unstable, lack species and host demarcations, and are not favored by evolution

**Fig 5:**
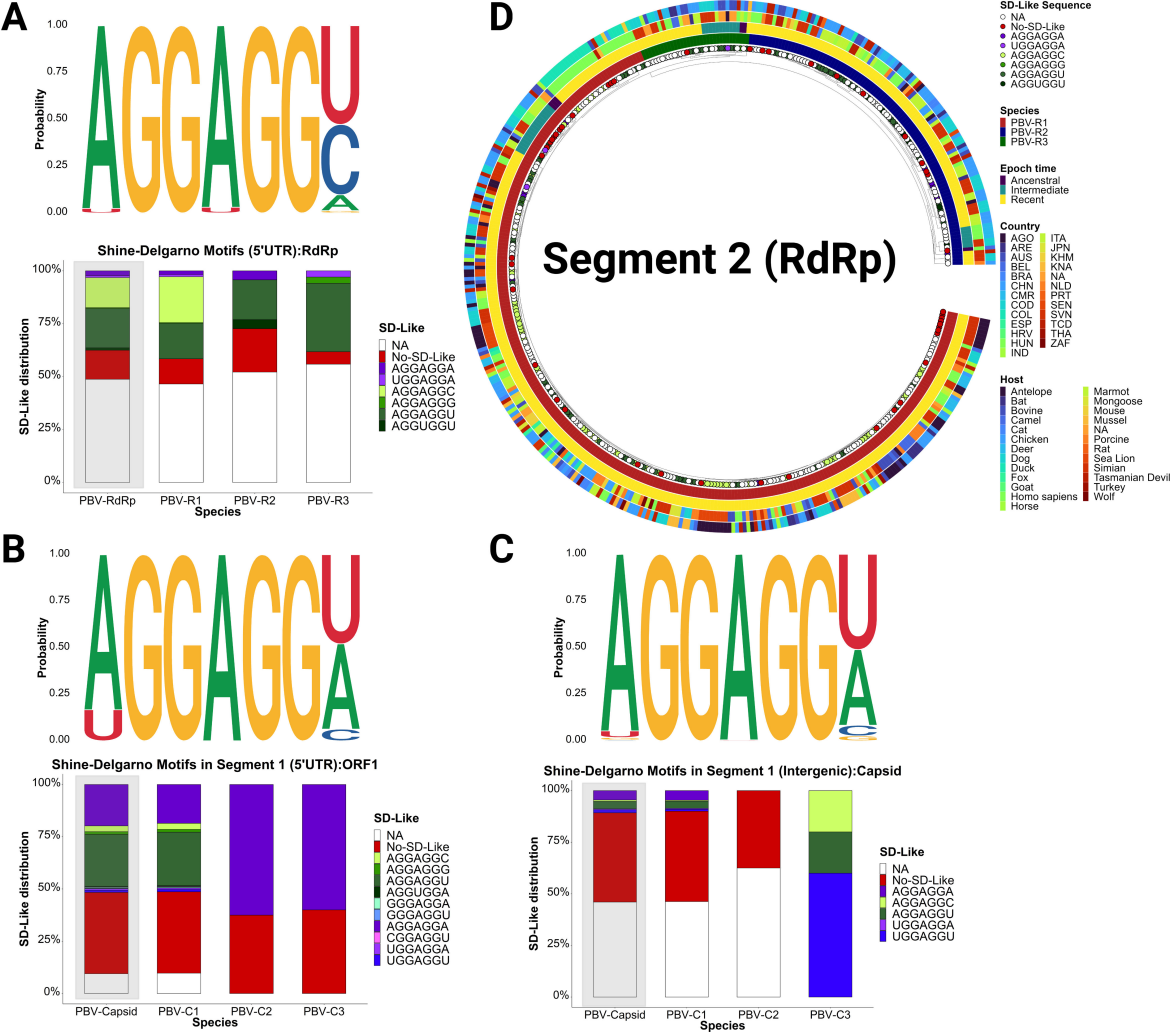
Distribution and variability of Shine-Dalgarno motifs on both Picobirnavirus genome segments. (A–C) Logo illustration of the nucleotide substitution rates expressed as probability of nucleotide base per position (top), with the combined distribution (%) of each sequence on both segments (light-gray) as well as by individual species (11) (bottom). (**D**) Temporal phylogenetic tree based on the coding region of segment 2 (RdRp) reconciled by the SD-Like motif (tips) distribution. Species demarcation (inner ring), Epoch time of emergence (second ring), stability of the SD-like sequence (determined by entropy of each position Fig. S5]) (third ring), host restriction (fourth ring), and geographic distribution (outer ringer) are all indicated in the phylogeny.

We therefore returned to the presence of Shine-Dalgarno (SD) sequences offered as evidence of PBV’s prokaryotic origins (5). Should these ribosome-binding signals indeed be functional within its bacterial host, one might anticipate a strict adherence to the AGGAGGU consensus, which has been previously reported with a curated set of examples (7). Here, we performed an exhaustive analysis of all available sequences (segment 1, *n* = 465; segment 2, *n* = 437) and observe far less conclusive results (Fig. 5). To begin, either most deposited strains lack this sequence information (e.g., N/A: 5′ end missing) or there is nothing resembling an SD consensus upstream of the coding region. For the latter case, 27% of 5′UTRs preceding segment 2 RdRp, 43% of 5′UTRs upstream of segment 1 ORF1, and 80% of intergenic regions upstream of capsid do not possess this motif. Remaining sequences displayed several variations of the SD-like element (Fig. 5). Stability of the SD consensus is required to act as a *cis* regulator of translation (17). However, we observed considerable variability both in the SD consensus and within the length of the requisite 5 ± 2 nucleotides preceding the ATG start codon (Fig. 5A through C; Fig. S4 and S5). Focusing on RdRp, while one might have expected greater conformity among the more ancestral PBV-R_2_ sequences, we failed to observe trends along species lines (Fig. 5D). The proportion of stable SD elements was similar between PBV species, and there was no correlation with the time of diversification, suggesting the acquisition of this sequence was a random event not primed by evolution. Finally, we explored the distribution of SD sequences in terms of reported host and geographic restrictions and again failed to observe a linkage, yet this admittedly does not preclude infections with bacteria harboring these PBV strains (17). For comparison, we analyzed 109 available segment L sequences encoding *RdRp* from the *Cystoviridae* family. The canonical SD motif AGGAGGG was present in 100% of the species analyzed without nucleotide variation (Fig. S6A and B; Table S14). We explored the secondary structure of the 5′UTR in bacteriophage phi6 and obtained the formation of several stem-loops resembling IRES-like structures which stabilize the SD region (Fig. S6C) and previously described as alterative translation strategy for this viral species (reviewed in reference 18). Experimental evidence in agreement with our findings is manifested in the inability of PBV to replicate in bacterial culture, although this one example is not conclusive of the lack of potential to do so, and future experiments may prove otherwise (8). The source of this motif in PBV is therefore unclear; however, we hypothesize that PBV-R2’s relatedness to PBV-like viruses and mitoviruses could have been acquired through their interaction with hosts like Jakobids that carry SD in the mitochondria (19, 20).

### Functional divergence directed changes in the RdRp of PBV-R_2_ to facilitate the capsid-nascent RNA-RdRp interaction

**Fig 6:**
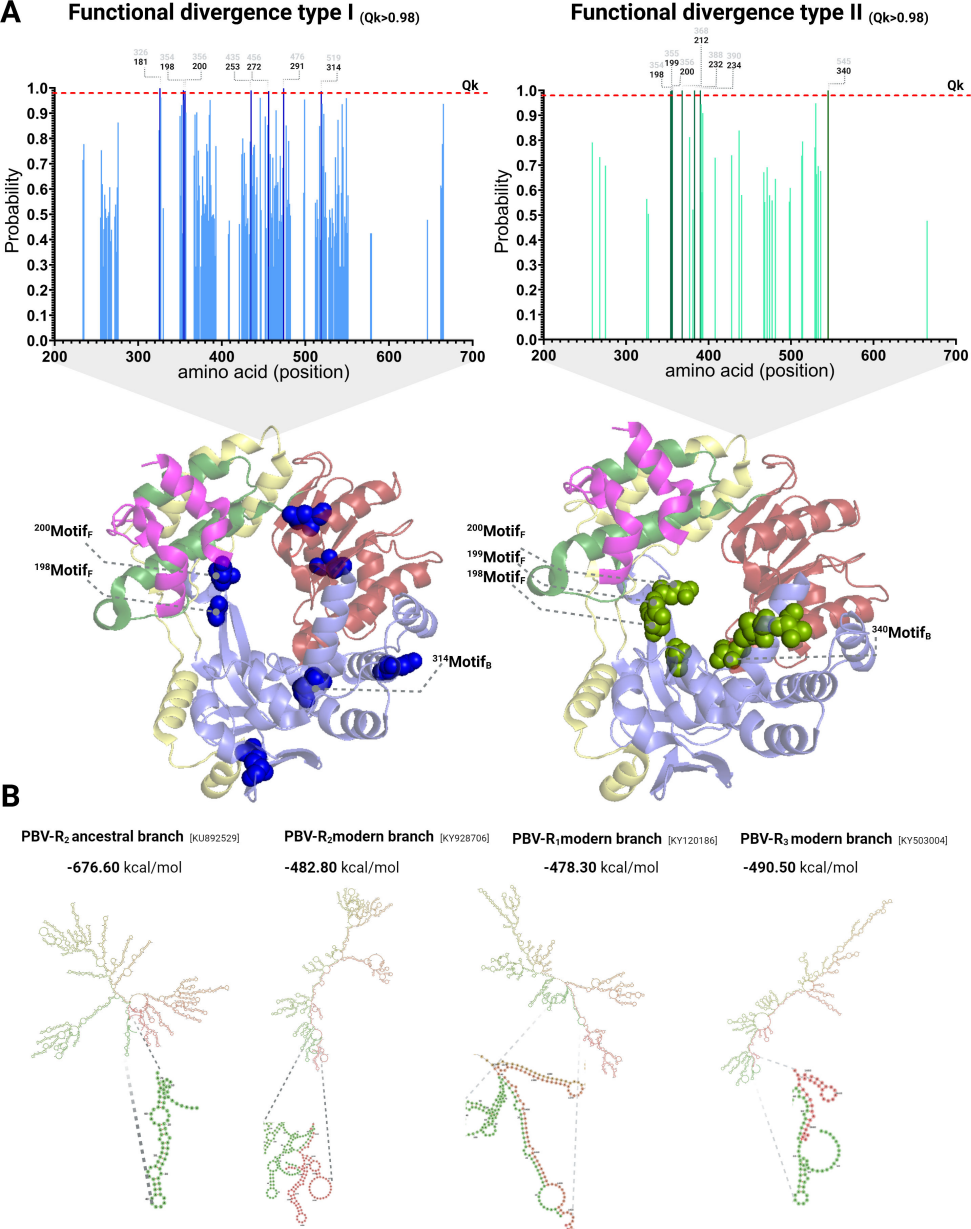
Functional divergence of the *RdRp* of PBV species. (A*,* top) Plots of amino acid residues with statistical support in RdRp that were changed by the action of functional divergence type I (in blue) and type II (green) (see Tables S7 and S8). Numbers in gray represent the AA position in the alignment and those in bold indicate positions after eliminating gaps, relative to the PBV-R2 reference sequence (accession number GU968924). Residues exceeding the posterior Bayesian statistics cut-off, Qk > 0.98 (red dashed line), were labeled with dark-blue (type I) and dark-green (type II) bars. (bottom) Schematic of 3D structure of human PBV-R1 *RdRp* (PDB ID: 5i62) with domains colored as in Fig. 3. Coinciding positions in PBV-R1 are with reference to accession number GU968924. Blue spheres labeled with positions and domain motifs indicate type I functional divergence sites and green spheres indicate type II functional divergence sites. (B) RNA secondary structures for the entire RdRp sequence from representative PBV strains were obtained using RNAfold. The Bayesian Integrated Coalescent Epoch PlotS (BICEPS) model identified PBV-R2/human/BEL/HPBV959/2010 (KU892529) as an ancestral strain and PBV-R2/Marmot-picobirnavirus/c330624/2013 (KY928706) as a contemporaneous strain. PBV-R1 and PBV-R3 sequences were analyzed for comparison. Minimum free energy (MFE) values for RNA secondary structures are listed and coloring of RNA molecule ends facilitated the visualization of the interactions between the 5′UTR (blue) and the 3′UTR (red).

If PBV-R_2_ indeed acquired its capsid from PBV-R_1_/R_3_, hallmark mutations or elements such as the SD sequence fixed after a duplication and reassortment process, which occurred between the two species would be expected. Indeed, the presence of non-segmented PBVs in Himalayan marmots suggests a mechanism by which homologous recombination occurs, mediated by GAAAGG direct repeats in the 5′UTRs of each segment (12). To explore this possibility, a functional divergence assessment was undertaken comparing the RdRp coding sequences. The results indicate that the coefficients of type-I functional divergence (θI) and type-II functional divergence (θII) between PBV-R_1_ and PBV-R_2_ species yielded statistically significant differences (Tables S3 and S4), whereas the other combinations interrogated (PBV-R_2_/PBV-R_3_ and PBV-R_1_/PBV-R_3_) were not supported. Residues identified after a site-specific posterior probability (Qk > 0.98) distribution analysis that exceeded the cutoff were represented as bold lines (upper panels) and mapped onto the 3D structure to identify which amino acids may have played a role in shifting the ancestral function of PBV-R_2_ (Fig. 6). Type I residues (left, blue) are highly conserved in PBV-R_1_ but are variable in PBV-R_2_, indicating they have different constraints. The implications are that mutations in PBV-R_2_ were selected for their ability to acquire the same functionality as in PBV-R_1_. Based on the RdRp crystal structure from Collier et al. (21), these amino acids are located primarily in motifs B and F which are known to mediate the interaction of RdRp with capsid during packaging (21). Type II residues (right, green) are biochemically distinct from one another in PBV-R_1_ vs PBV-R_2_ but highly conserved within in each protein. These residues may have different properties but the same function, or they can promote differentiation in function of the proteins. Once again, these amino acids are located in the very same region of *RdRp* as those detected by type I functional divergence. The results strongly suggest these mutations in PBV-R_2_ were essential for its ability to interact with capsid and consequently its ability to infect mammals.

### The genomic RNA of PBV-R_2_ evolved to a more stable and compact structure to enable encapsidation

To prevent triggering an immune response, dsRNA viruses need to accomplish replication and transcription within the confines of the capsid (22). A 120-subunit icosahedral architecture is another hallmark of dsRNA viruses (23). These functional and space constraints inevitably restrict the pairing of incompatible capsids and RdRps. The encapsidation process requires the presence of genomic RNA, mediating the indirect interaction between capsid and RdRp through the AU-rich 5′UTR stem loops in both segments (21). During assembly, the 5′UTR is bound by the flexible N-terminal region of the capsid protein (10, 21). RdRp motifs B and F play the most relevant roles in the stability of both template and nascently transcribed RNA, with motif B facilitating strand selection and passing the new (+)RNA to the palm and fingers and motif F involved in RNA synthesis via the movement of the rNTP. Residues identified by our functional divergence analysis were located in these critical RdRp domains and likely guarantee the correct interaction with the capsid via the RNA intermediate (Fig. 6A). Unlike partitiviruses containing only one genomic segment per virion, PBV particles package both genomic segments, as well as the viral polymerase bound to each (23). Therefore, upon acquisition of a capsid, PBV-R_2_ would require a shift in biochemical or charge properties of these functional residues to ensure replication and transcription of two segments and accommodate the protein-RNA-protein interaction needed for encapsidation. Based upon the time-heterogenous analysis, we selected contemporaneous strains of all three species and the most ancestral strain of PBV-R_2_ to compare the RNA secondary structures and free energies (Fig. 6B). The folding pattern of the ancient PBV-R_2_ had a higher minimum free energy compared with its more recent progeny, with the latter exhibiting a more compact, elongated structure for packaging. Moreover, the key distinction is the evolution away from a self-folding 5′UTR to the complementary base pairing of the 5′UTR and 3′UTR. Remarkably, equivalent structures and free energies were observed in PBV-R1 and PBV-R3 (Fig. 6B), underscoring that the annealing of termini was evolutionarily favored upon acquisition of the capsid.

## DISCUSSION

Recent advances in sequencing and genomic surveillance have highlighted the potential for zoonotic transmission of Picobirnavirus, yet determining the true origin of these viruses has remained elusive (5). Approaches using structural biology (10, 21) and morphogenesis (23) have highlighted their resemblance to both *Reoviridae* and *Partitiviridae* families. Here, by combining temporal, statistical, and integrated phylogenetic analyses, we reveal that both sides of the debate appear to be correct. PBV emerged from different ancestral vertebrate and fungal hosts, and it was through parallel adaptation that they converged to become the viral phenotypes of the present day.

Parallel molecular evolution is a widespread and important phenomenon in viral species adaptation (24, 25). The absence of capsid from the most ancestral species (Fig. 1), along with the dual emergence of RdRp from different dsRNA viral families (Fig. 2), provided evidence that PBV evolution was being driven by parallel patterns of fitness improvement. The adaptive divergence (also known as selectionism) (26) (Fig. 4) displayed by PBV-R_2_ upon acquisition of the capsid, combined with the subsequent functional divergence (as evidence of mutationism) (26) of its genomic RNA and RdRp domains to facilitate encapsidation (Fig. 6), also stood as clear examples that this pivotal event shaped PBV evolution. Thus, capsid folding and assembly appear to impose a selective constraint which drove the convergence of *Picobirnaviridae* family members. In Monttinen et al., structure-based clustering of 120 subunit icosahedral capsids correlated with RdRp phylogenies which led the authors to surmise that PBV RdRp and capsid genes descended from cystoviruses or reoviruses (27). Indeed, our tree showed this relationship (Fig. 2) and recall many additional parallels with *Reoviridae*. The proteolytic maturation of PBV capsid protein is similar to cleavage in orthoreovirus capsid mu1 (10). The T1 innermost core in reoviruses (e.g., blue tongue virus) resembles the PBV capsid structure and acts as a scaffold to prime assembly of the surrounding T13 capsid needed for cell entry. The five pores in PBV used to release mRNA during transcription also mimic Reovirus. PBV presumably requires a mono-particulate virus (both segments packaged in the same virion) to enable transmission to neighboring cells during an extracellular phase of its life cycle (23). Thus, its viral particle is tightly packed and has the same density as rota-/reoviruses (34–38 bp/100 nm^3^). By contrast, partitiviruses (fungal) are loosely packed (20 bp/100 nm^3^), with only one segment per virion, despite multi-segmented genomes (23).

Our dating of PBV emergence roughly 600 years ago is not consistent with the RNA bacteriophage evolutionary hypothesis, since the latter lack a molecular clock (28). Evolutionary rates of 10^−3^ substitution/site/year, the phylogenetic reconstructions obtained, and the apparent lack of a regulatory role for Shine-Dalgarno signals (Fig. 5) suggest picobirnaviruses are not prokaryotic viruses. In a recent study, Boros et al. (8) claimed functionality of SD sequences in PBVs; however, controls such as mutating or deleting SD sequences from expression cassettes were not performed, and therefore, expression could be due to non-canonical translation-initiation mechanisms in *E. coli* (e.g., leaky translation [29]). The high degree of SD conservation and IRES-like secondary structure in *Cystoviridae* suggesting a *cis* regulatory role contrasts with the sporadic and variable presence of the SD and RNA base pairing patterns observed in *Picobirnaviridae* (Fig. 6B; Fig. S6).

For PBV-R_2_, its prior lack of a capsid and relatedness to PBV-like and mitoviruses certainly suggest these species resided in lower organisms, were not under selective immune pressure, and are comparatively more ancient viruses. Structural and phylogenetic relationships to partitiviruses raise the possibility that PBV-R_2_ crossed the species barrier from unicellular eukaryotic organisms to vertebrates. By contrast, PBV-R_1_/R_3_ phylogeny indicates they originally descended from Reovirus ancestors and may have emerged from a vertebrate host. Our findings point to convergent evolution as having driven the similarities we see shared today among these three PBV species. Interestingly, PBV has not evolved to impose host restrictions, as strains cross vertebrate lines and the same virus is able to infect different species (e.g., pig and human) (30, 31). However, new mutations may alter cellular tropism and drive gastrointestinal strains to emerge as respiratory pathogens (32). As unsampled ancestors get sequenced and new techniques are developed (cell culture model), we will inch closer to an answer, but our study suggests that PBV species were derived from distinct host origins and provide a clear example of parallel evolution mechanisms at work.

### Conclusions

Our study reveals that PBV species started from two distinct origins and that convergent evolution drove the diversification and evolution of *Picobirnaviridae*. Epistatic interactions, functional divergences, and positive selection synergistically favored the acquisition of the capsid in the most ancestral species, the pivotal event that shaped the dynamics of the entire family. Their contemporaneous emergence, together with the frequent absence and heterogenous nature of the SD which suggests these are non-functional, evolutionary vestiges, argues against a prokaryotic host. Rather, the timing of the accumulation of mutations in PBV-R2 that promoted encapsidation is consistent with a host-jump event requiring adaptation to an extracellular lifestyle in a vertebrate host. These insights provide new dimensions to the PBV origin and pathogenicity debates.

## MATERIALS AND METHODS

### Sequence data set, alignments, and phylogenetic analysis

Different sequence data sets were created for different purposes in this study, including temporal inference, phylogenetic, divergence, and coevolutionary analyses. Data sets A and B initially contained all Picobirnavirus genus sequences for both capsid and *RdRp* coding regions, respectively. All sequences utilized in Perez et al. (33) and Berg et al. (3) with defined isolation times were included into these two datasets. Sequences were downloaded from GenBank (http://www.ncbi.nlm.nih.gov/) on 1 August 2021 and filtered as described in Perez et al. (33) (Table S5 and S6). Data set C was created to infer the phylogenetic relationship among all PBV-related double-stranded RNA viral families. It contains the *RdRp* coding sequences previously described in Knox et al. (13) as well as those from the PBV-like group (9) omitted in Knox et al. (13). All 640 sequences included in data set C were downloaded from GenBank on 20 January 2022 and filtered as above (Table S7). Finally, data sets D, E, and F contain sequences from each PBV species defined in Perez et al. (33) and were created for the adaptive divergence and coevolutionary analyses (Tables S8 to S10).

All data sets for both nucleotides and deduced amino acid sequence alignments (MSA) were created using Multiple Alignment using Fast Fourier Transform (MAFFT) with the option E-INS-I to decrease the penalty in the gaps as described in Perez et al. (33) Phylogenetic analyses using maximum likelihood inferences were performed as described in Perez et al. (34) Briefly, ML phylogenetic trees derived from either nucleotides or deduced amino acid alignments were computed with the IQ-TREE2 program (35) using the ModelFinder algorithm to select the best-fit model for each data set. Confidence levels for branches and internal nodes were determined by 1,000 replicates of both the Shimodaira-Hasegawa approximate likelihood ratio test (36) and ultrafast bootstrapping (37) to each phylogenetic inference. Trees were used as input files for the temporal Bayesian analysis, edited, and visualized using Figtree and R-script (GitHub repository).

### Molecular clock test, Bayesian model and prior selection, and temporal analysis

The presence of the temporal signal in data set A was initially tested by two methodologies: (i) the regression of the distance from the root to each of the tips as a function of the sequence sampling times, known as root-to-tip regression, and (ii) an explicit assessment of the temporal signal using the Bayesian evaluation of temporal signal test (38). The former was determined using TempEst (39) and TreeTime (40) to calculate the underlying temporal signal using the heuristic residual mean squared and least squares models, respectively, whereas BETS was conducted using BEAST v.1.10.4 (41). BETS statistically contrasts two competing models (one in which the data are accompanied by the actual sampling times [heterochronous] and the other one in which the sampling times are constrained as contemporaneous [isochronous]). Thus, if the heterochronous model improves the statistical fit of the data, the use of a molecular clock to calibrate the data is warranted (38). The estimation of the marginal likelihood (*M*) of these two competing models (heterochronous and isochronous) was achieved with the estimators of log marginal-likelihood (log *M*): path sampling (42) and stepping-stone sampling (43). The analysis was performed as recently described by Orf et al. (44) with some modifications, briefly: two molecular clocks were tested: a strict clock (SC) and an uncorrelated relaxed lognormal clock (45), with an exponential distribution prior with a mean of 1.0 and a constant coalescent prior using an exponential population size with a mean of 10.0 and offset of 0.5. The analyses were run using BEAST 1.10.4 (41) with a chain length of 5 × 10^8^ interactions, from which a 10% burn-in was discarded. Thus, four total runs (one for each hypothesis with each clock prior) were conducted per genome segment. In all cases, the marginal likelihood estimation (MLE) using path sampling/stepping-stone sampling was set to a 5 × 10^6^ length of chains with 500 number of path steps and the default beta distribution of the path step (46, 47). Comparison of the statistics from both the path sampling and stepping-stone sampling methods was tabulated to compare the models (Table 1).

Likewise, the molecular clock model and the prior that best fit the analyzed data set were selected based on the results of these two estimators (PS and SS) as described in De la Cruz et al. (48). For both genomic segments, an uncorrelated relaxed local clock with a Bayesian skyline plot as a prior was selected (Tables S1 and S2). The initial ML trees estimated by IQ-TREE2 were used as starting trees in the temporal analyses by the Bayesian approach. For both genomic segments, two Markov chain Monte Carlo (MCMC) chains were run over 200 million states using BEAST v1.10.4 (49) with sampling every 20,000 states. Both chains were combined after 10% of states were removed (burn-in step). For the RdRp and capsid sequences, WAG+gamma (four categories) and LG*+*gamma (four categories) substitution models were used, respectively, together with the previously selected molecular clock and prior. For both segments, we used a CTMC scale prior for the rate and an uninformative uniform distribution for the skyline.popSize since there is no previous evolutionary information about PBVs. Convergence for both MCMC chains and parameters was assessed using Tracer v1.7 (50).

### Adaptive divergence analysis

The adaptive divergence analysis was performed as described by Kistler and Bedford (51) with minor modifications. First, nucleotide alignments for data sets D, E, and F (Tables S7 to S9) were used to infer time-heterogeneous phylogenies using a BICEPS model (52). Three epochs with windows of 50 years was applied to each viral species, with epoch times demarcating when statistical differences were observed in the kappa (*κ*_*n*_) values (defined as the transition-transversion bias values mutational ratio bias]). Two Markov chain Monte Carlo chains were run over 400 million states using BEAST 2.6.3 (53) with sampling every 40,000 states. Both chains were combined after a 10% burn-in, and MCC trees and *κ*_*n*_ values for each epoch time were obtained. Second, to measure episodic selection, non-synonymous/synonymous mutation rate ratios (*d_N_*/*d_S_:ω_2_*) for each internal node of the time-heterogeneous phylogenies were determined as described by Perez et al. (34). Briefly, the CODEML program of the PAML v.4.9 software package (54) was used applying a branch-site model (A and A_1_) to pre-specified branches (hypothesized under positive selection [foreground branches]) in comparison with the remaining branches (background branches). The branch-site model was tested under the null (*ω* along all branches [0 < *ω*_1_ < 1 and *ω*_2_ = 1]) and alternative (*ω*_2_ > 1) (54), and both hypotheses were contrasted by likelihood ratio tests (LRTs). The significance of the LRTs was estimated assuming a Chi-square (*χ* ^2^) distribution with degrees of freedom assigned as the difference in the number of parameters in the two types of models, as previously determined in Perez et al. (34). In all cases, a *ω*_2_ = 1 assumes that significant differences were not found between the branches (Tables S11 to S13). Finally, the adaptive divergence for each species was determined by adding the ratio (window of 50 years) obtained from the weighted episodic selection with the ratio of mutational bias during the epoch time in which each lineage was assessed (Tables S11 to S13).

### Coevolution analysis

Some amino acids (due to their composition and location) can affect the function and the stability of a protein more than others (55), and substitutions at these “relevant” positions can occur once compensatory changes take place in the protein (56). Coevolutionary analyses can identify these structural and/or functional interactions (55) and thus was conducted to reveal compensatory substitutions in RdRp of different PBV species, likely in response to host constraints (immune response and/or adaptation). As the number of sequences available for each species differed considerably (see data sets D, E, and F), the recently developed algorithm BIS2TreeAnalyzer (57) was employed since it was specifically designed to identify clusters of coevolving residues in alignments with a relatively low number of sequences (58). MSA of the deduced amino acid sequences of the data sets D, E, and F together with their respective phylogenetic trees were used as input files. The most ancestral sequences identified in the time-heterogeneous phylogenetic analysis were selected as roots for each tree. Taking into account intra-species variability in the *RdRp* sequence for each PBV species (33), an alphabet reduction by physicochemical properties was set. A coevolutionary dependency network for each intra- and inter-*RdRp* domain was constructed based on the parameters described by Champeimont et al. (15), and a weight defined as the sum of interactions (direct and indirect [see Champeimont et al. {15} for definition]) was summarized and visualized using the R package circlize (59).

### Functional divergence analysis

For functional proteins like *RdRp*, replacement rates of amino acids are heterogeneously distributed across the entire sequence and linked to the natural selection (60). Thus, type I functional divergence uncovers those differential rates (acceleration or deceleration) in each amino acid position known as “covarion”-type model (61) and type II unveils the changes in the physicochemical properties among conserved residues of two different groups, known as constant-but different mode) (62). DIVERGE software (version 3.0) (63) was used to measure type I and type II functional divergence. Coefficients θ*I* and θ*II* among PBV-R_1_, PBV-R_2_, and PBV-R_3_ were estimated to test the statistical support of the analyses (64), and a site-specific posterior probability (Qk) analysis was applied to predict which amino acid residues were crucial for functional divergence. High Qk values indicate an elevated degree of the functional constraint or that the change in the residue property at a site is different between two clusters. Based on the distribution obtained, the critical value of Qk was set to 0.98.

### Shine-Dalgarno-like motifs characterization

To determine the role of the Shine-Dalgarno-like motifs (SD-like) in *Picobirnaviridae*, all nucleotide sequences for both segment in the current study (Tables S5 and S6) were individually inspected using SnapGene software (www.snapgene.com) to accurately separate the coding region from the untranslated region. Thus, the 5′UTR for both segments 1 and 2 was extracted as well as the upstream untranslated region of the capsid (hereafter denoted as intergenic). All extracted sequences were aligned using Bioedit (65) and visually inspected for the canonical Shine-Dalgarno sequence AGGAGGU, allowing degrees of variability surrounding the core xGGxGGx. The software Metalogo (66) was used to identify group distribution and stability based on entropy. The R-package ggseqlogo was used to identify distribution proportion of the SD-Like element for each segment based on probability (67). Distribution percentages of SD-like sequences were visualized as stacked histograms using the Tidyverse R-package (68). Finally, a reconciliation between the composition of the SD-like sequence motifs with the species demarcation, epoch time (determined in the section above), stability measured by entropy (Fig. S6), host restriction, and geographic distribution was mapped by using the R-package ggtreeExtra (69). For comparison purposes, the dsRNA phage family *Cystoviridae* was selected to evaluate the presence and conservation of SD-like motifs. From the 608 sequences available at the GenBank database on 21 June 2023, the 109 complete sequences for the L segment encoding for the *RdRp* of the different species of this viral family were downloaded (Table S14). The untranslated region was inspected for the presence of SD-like motifs as described above. In addition, the secondary structure of the 5′UTR of all 62 sequences for the bacteriophage phi6 on which the translation mechanism has been previously described was determined as described below (“RNA secondary structure”), in order to visualize a model of the putative role the SD-motif in the translation process.

### RNA secondary structure

The RNA secondary structures for the entire segment 2 (RdRp) of different PBV species were obtained using the RNAfold Webserver on 28 September 2022. PBV-R_2_/human/BEL/HPBV959/2010, PBV-R_2_/Marmot-picobirnavirus/c330624/2013, PBV-R_1_/Simian/PBV/13R/2009, and PBV-R_3_/Variants-V39/Gorilla/2015 (accession numbers KU892529, KY928706, KY120186, and KY503004, respectively) were selected as representatives. For PBV-R_2_, KU892529 was identified as ancestral and KY928706 was selected as contemporaneous by the time-heterogeneous phylogeny analysis. Contemporaneous PBV-R_1_ and PBV-R_3_ strains were used for comparison. Also, the secondary structure of the 5′UTR of Phi6 (Table S14) was determined. The folding of secondary RNA structures was computed as described by Relova et al. (70). Briefly, MFE parameters, equilibrium base pairing probabilities, and the partition function (PF) were selected to obtain folding patterns. Pairing probabilities were visualized on the RNAfold Webserver using the forna format (http://rna.tbi.univie.ac.at/forna/forna.html) with colors set by position to facilitate the visualization of the interactions between the 5′UTR and the 3′UTR of the RNA molecule.

## Data Availability

All the raw data, including multiple sequence alignments and phylogenetic trees, obtained during the execution of the current study are available in the public repository below. The R-scripts used for data visualization and analyzing the effects of taxon sampling are also available in the same repository: https://github.com/LesterJP/PBV_Research.

## SUPPLEMENTAL MATERIAL

Supplemental material spectrum.02693-23-s0001.pdf Tables S1 and S2, Fig. S1 to S7, Tables S3 and S4, and Tables S11 to S13.

Table S5 spectrum.02693-23-s0002.xlsx Data set A sequences of PBVs.

Table S6 spectrum.02693-23-s0003.xlsx Data set B sequences of PBVs.

Table S7 spectrum.02693-23-s0004.xlsx Data set C sequences of RdRp segment for dsRNA viruses.

Table S8 spectrum.02693-23-s0005.xlsx Data set D sequences of RdRp segment of PBVR1.

Table S9 spectrum.02693-23-s0006.xlsx Data set E sequences of RdRp segment of PBVR2.

Table S10 spectrum.02693-23-s0007.xlsx Data set F sequences of RdRp segment of PBVR3.

Table S14 spectrum.02693-23-s0008.xlsx Cystoviridae sequences used as control for Shine Delgarno stability evaluation.

ASM does not own the copyrights to Supplemental Material that may be linked to, or accessed through, an article. The authors have granted ASM a non-exclusive, world-wide license to publish the Supplemental Material files. Please contact the corresponding author directly for reuse.
