# Supplementary material for: Parallel evolution of picobirnaviruses from distinct ancestral origins
Source: Microbiol Spectr. 2023 Oct 27;11(6):e02693-23. doi: 10.1128/spectrum.02693-23 (PMC10714727; doi:10.1128/spectrum.02693-23)
Supplement: Supplemental material — Tables S1 and S2, Fig. S1 to S7, Tables S3 and S4, and Tables S11 to S13. [file spectrum.02693-23-s0001.pdf]

Supplementary information

Parallel evolution of Picobirnaviruses from distinct ancestral origins

Lester J. Perez[a,b]*, Gavin A. Cloherty[a,b], Michael G. Berg[a,b]

TableS1. Bayesian model selection based on path-sampling(PS) and stepping-stone (SS) algorithms for RdRp coding sequences of Picobirnavirus.

**Clock Model**	**Coalescent**	**Path-Sampling (PS)**	**Stepping-Stone (SS)**
Strict	Constant	-176874.88	-176894.6043
	Exponential	-176798.1118	-176818.2978
	Bayesian Skyline Plot	-176580.2855	-176567.3726
	Logistic	-176797.1118	-176597.3726
**Uncorrelated Relaxed Local**	Cosntant	-176197.8493	-176197.8493
	Exponential	-176114.8604	-176128.0448
	**Bayesian Skyline Plot**	**-175969.1107**	**-175969.1107**
	Logistic	-176114.8604	-176128.0448
Relaxed Local clock	Constant	-177565.457	-177557.0007
	Exponential	-176841.665	-176812.4936
	Bayesian Skyline Plot	-176386.745	-176625.274
	Logistic	-176596.745	-176405.274
Fixed Local clock	Constant	-176562.4866	-176583.5461
	Exponential	-176521.8566	-176545.4361
	Bayesian Skyline Plot	-176511.1366	-176525.4261
	Logistic	-176426.1366	-176385.4261

TableS2. Bayesian model selection based on path-sampling (PS) and stepping-stone (SS) algorithms for the capsid coding sequences of Picobirnavirus.

**Clock Model**	**Coalescent**	**Path Sampling (PS)**	**Stepping Stone (SS)**
Strict	Constant	-406058.9556	-406084.4724
	Exponential	-405836.2061	-405853.0488
	Bayesian Skyline Plot	-405357.6929	-405397.271
	Logistic	-405754.4413	-405778.9723
**Uncorrelated Relaxed Local**	Cosntant	-405793.8496	-405822.0878
	Exponential	-405557.0454	-405576.594
	**Bayesian Skyline Plot**	**-405085.058**	**-405116.8626**
	Logistic	-405516.3824	-405540.7029
Relaxed Local clock	Constant	-413164.3458	-413161.7727
	Exponential	-410854.8501	-410729.2901
	Bayesian Skyline Plot	-407335.6813	-407324.5291
	Logistic	-411322.2594	-411220.834
Fixed Local clock	Constant	-406015.4907	-406044.3634
	Exponential	-405873.5531	-405892.1387
	Bayesian Skyline Plot	-405429.25	-405464.2539
	Logistic	-405698.6964	-405734.1933

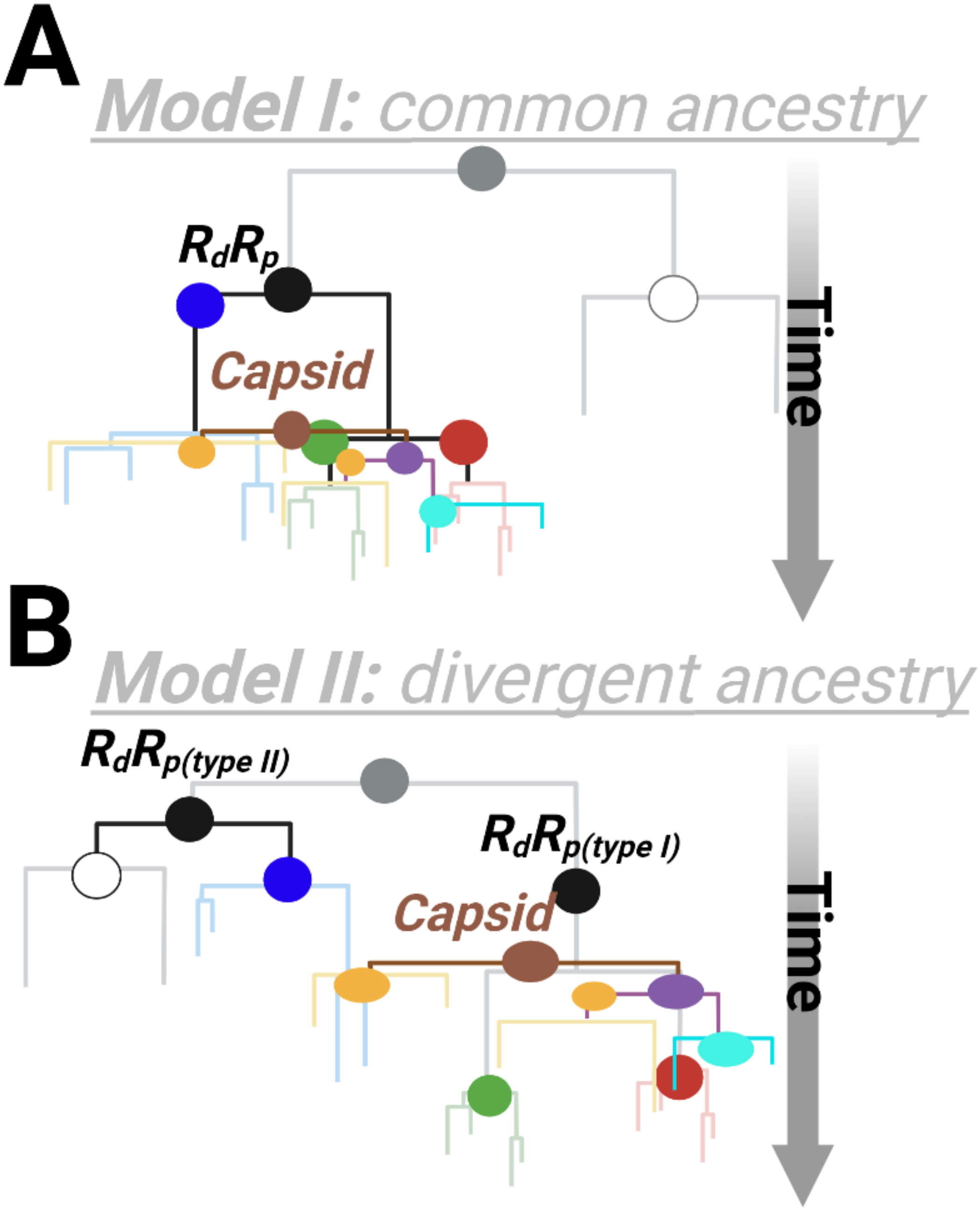

Figure S1. Theorized models for the putative evolution of the PBVs genome and reassortment events that can explain the discrepancies for the time of the most recent common ancestor (tMRCA) obtained for both viral segments. A) Model I describes the emergence of the segment encoding *RdRp* of all three PBV species from a common ancestor that further diversified into three different species and acquired the segment encoding for the capsid during this process of diversification as a result a reassortment event. B) Model II describes the emergence of the segment encoding *RdRp* for the PBV species from different ancestors with PBV-R_1 and PBV-R_3 having the segment encoding for the capsid since their emergence and PBV-R_2 acquired this segment later as a result a reassortment event with one these two species.

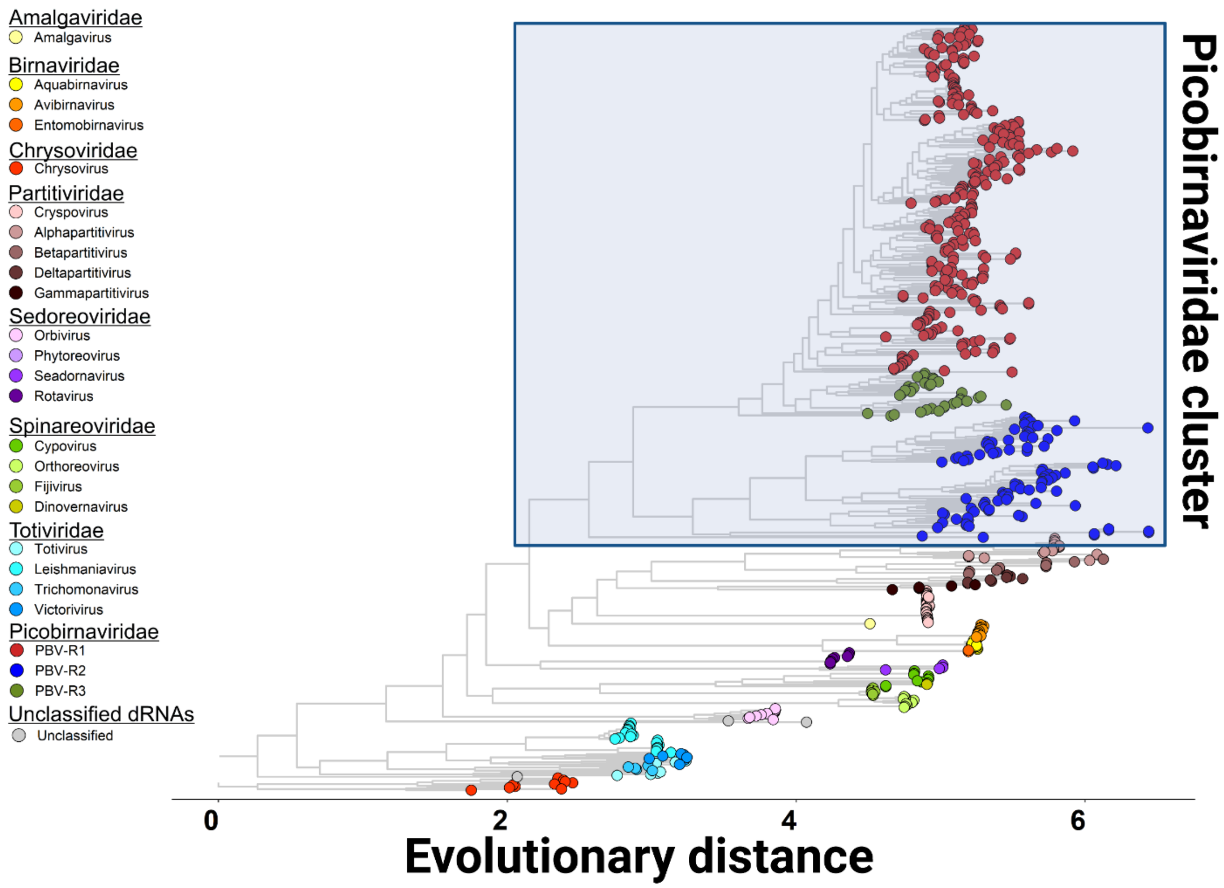

Figure S2. Resolved phylogenies for *Picobirnavirus* and closely related viral families after removing PBV-like viral sequences.
ML-tree using the complete coding region for the *RdRp* as described by Perez et al. (2021) for all sequences of PBVs and all *RdRp* available sequences at GenBank dataset for other dsRNA viral families closely related to PBVs as previously determined by Knox et al. (2018) (see supplementary material Table S3) were analyzed after removing the PBV-like viral sequences. Each viral family is denoted. The clustering patter of the *Picobirnaviridae* family groping as an individual cluster is denoted. This analysis reveals the impact of the taxon sampling on the resolution of phylogenetic analyses.

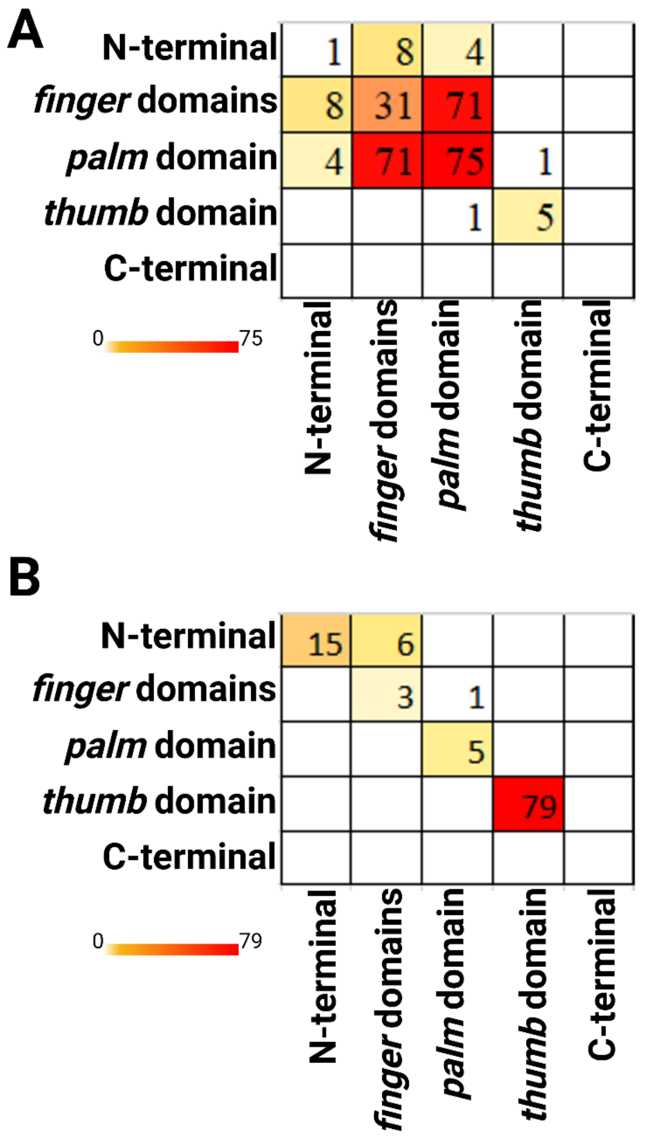

Figure S3. Matrices describing PBVs coevolving residues within the domains of the *RdRp* protein. The matrices describe the coevolution links for genotypes A) PBV-R$_1$ and B) PBV-R$_3$, the numbers indicate the count of coevolving residues (hits) identified in the clusters predicted by BIS2 analysis.

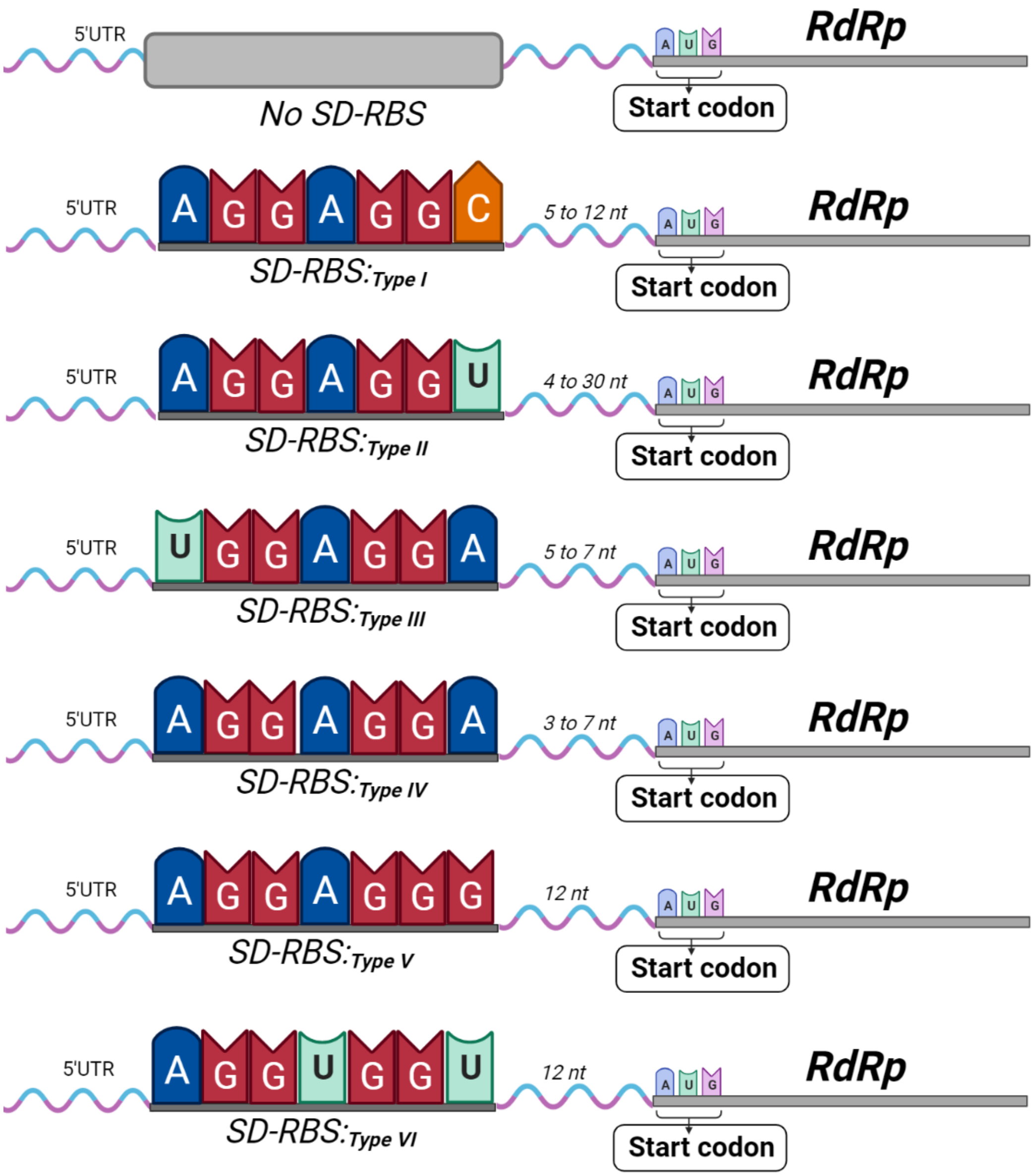

Figure S4. Shine-Delgarno motifs composition and distance to the start codon in the segment 1 of PBVs. The different nucleotide composition for the SD-Like in segment 1 is represented. The distance at which these motifs were found on the sequences analyzed (supplementary TableS9) was also denoted.

Table S3. Statistics evaluation for Functional Divergence type I (θ_I)

Species cluster	θML	θSE	θLTR	Log-score(alt)	Test
PBVR2/PBVR1	0.581	0.09954	70.607	5.838800092	24.18544012
PBVR2/PBVR3	0.0189	0.13261	0.005	0.142143999	0.070351194
PBVR3/PBVR1	0.001	0.06821	0	0.014661467	0

Table S4. Statistics evaluation for Functional Divergence type II (θ_{II})

Species cluster	θML	θSE	θLTR	Log-score(alt)	Test
PBVR2/PBVR1	0.028002	0.334608	1.260398	0.083685985	30.12208085
PBVR2/PBVR3	2.133938	1.157283	3.64684	1.843920631	3.95552817
PBVR3/PBVR1	0.131098	0.151317	0.572327	0.866379852	1.321191851

TableS5. Dataset A sequences of PBVs (Excel file)

TableS6. Dataset B sequences of PBVs(Excel file)

TableS7. Dataset C sequences of *RdRp* segment for dsRNA viruses (Excel file)

TableS8. Dataset D sequences of RdRp segment of PBVR1(Excel file)

TableS9. Dataset E sequences of RdRp segment of PBVR2(Excel file)

TableS10. Dataset F sequences of RdRp segment of PBVR3(Excel file)

TableS11. Estimation of adaptive divergence for each branch of the topological distribution of PBV-R_1

Branch No.	ω2	-lnL$^{n.m}$	-lnL$^{a.m}$	2ΔlnL	kappa	Divergence
1	1	20675.42035	20665.09622	**20.648272**	23.86	0.041911148
2	1	20673.29479	20664.79139	**17.006792**	23.86	0.041911148
3	1	20653.85223	20701.99799	**96.291514**	23.86	0.041911148
4	1	20658.66599	20695.46777	**73.603544**	23.86	0.041911148
5	1	20654.90432	20670.86648	**31.92432**	23.86	0.041911148
6	1	20649.50948	20732.20923	**165.399486**	23.86	0.041911148
7	1	20670.07100	20720.98491	**101.827818**	23.86	0.041911148
8	17.63435	20655.34133	20670.69759	**30.712506**	23.86	0.739075859
9	8.82166	20664.60959	20546.66964	**235.879916**	4.08	2.162171569
10	12.45272	20677.59830	20460.77798	**433.64065**	4.08	3.052137255
11	13.29739	20680.46336	20432.39453	**496.137672**	1.97	6.749944162
12	12.97539	20677.59830	20454.59316	**446.010294**	1.97	6.586492386
13	5.24163	20637.99753	20411.60804	**452.778984**	1.97	2.660725888
14	12.89243	20678.33349	20455.30493	**446.057126**	1.97	6.544380711
15	8.29829	20687.97740	20244.85065	**-886.253496**	1.97	4.212329949
16	7.3569	20674.51936	20383.81932	**581.40008**	1.97	3.734467005
17	5.75558	20677.87377	20319.06919	**717.60915**	1.97	2.921614213
18	7.38811	20677.59830	20438.74328	**477.710044**	1.97	3.750309645
19	7.5836	20677.59838	20277.82429	**799.54818**	1.97	3.849543147
20	12.67451	20677.59833	20454.16592	**446.864836**	1.97	6.433761421
21	11.82566	20678.33349	20452.91092	**450.845152**	1.97	6.002873096
22	7.54621	20677.59830	20356.69311	**641.81039**	1.97	3.830563452
23	11.29388	20680.46332	20415.39545	**530.135742**	1.97	5.73293401
24	11.29268	20678.33349	20538.56516	**279.536672**	1.97	5.732324873
25	9.72481	20681.13057	20579.53572	**203.189688**	1.97	4.936451777
26	11.98452	20677.57426	20538.36004	**278.42843**	1.97	6.08351269
27	15.9566	20675.44488	20537.62332	**275.643116**	1.97	8.099796954
28	13.30203	20675.44488	20560.58026	**229.729244**	1.97	6.752299492

lnL: log-likelihood scores; n.m: null model; a.m: alternative model; **$p<0.01$, $\chi 2= 5.99$.

TableS12. Estimation of adaptive divergence for each branch of the topological distribution of PBV-R_2

Branch No.	ω_2	$-lnL^{n.m}$	$-lnL^{a.m}$	2ΔlnL	kappa	Divergence
1	1	13866.4354	13875.18425	**17.497692**	30.94	0.032320621
2	1	13882.67565	13884.19287	3.034424	3.872	0.258264463
3	1	13885.34852	13883.41785	3.86133	3.872	0.258264463
4	1	13890.83911	13889.10229	3.473628	3.872	0.258264463
5	9.43377	13835.63348	13887.21133	**103.155708**	1.5	6.28918
6	9.79305	13851.90574	13883.97907	**64.146664**	1.5	6.5287
7	13.93214	13829.94186	13888.46807	**117.052416**	1.5	9.288093333
8	1	13886.73613	13889.32072	5.169188	1.5	0.666666667
9	1.3517	13870.87184	13889.32072	**36.897764**	1.5	0.901133333
10	1.62618	13867.04395	13886.30752	**38.527138**	1.5	1.08412
11	5.4619	13820.16204	13888.26800	**136.211922**	1.5	3.641266667
12	1	13875.76069	13885.15230	**18.783208**	1.5	0.666666667
13	7.23057	13868.76033	13884.09239	**30.664122**	1.5	4.82038
14	1	13865.25062	13887.18311	**43.864974**	1.5	0.666666667
15	8.37604	13779.00302	13850.25193	**142.497814**	1.5	5.584026667
16	1	13882.55496	13889.32072	**13.531528**	1.5	0.666666667
17	16.42069	13793.34936	13885.91041	**185.122096**	1.5	10.94712667
18	8.65692	13801.99275	13871.05950	**138.133506**	1.5	5.77128
19	1	13886.11633	13886.17421	0.115766	1.5	0.666666667
20	53.45107	13807.79151	13884.64706	**153.711102**	1.5	35.63404667
21	30.37265	13829.34715	13884.73869	**110.78309**	1.5	20.24843333
22	1	13889.87872	13887.60752	4.542398	1.5	0.666666667
23	1.42816	13853.77959	13885.13215	**62.705106**	1.5	0.952106667
24	1.63968	13836.79291	13880.14203	**86.698234**	1.5	1.09312
25	1.55117	13875.24567	13889.02954	**27.56774**	1.5	1.034113333
26	1.42987	13877.18184	13884.50996	**14.656234**	1.5	0.953246667
27	1.68024	13866.4354	13884.19617	**35.521548**	1.5	1.12016
28	1	13885.26931	13884.19617	2.14628	1.5	0.666666667
29	1	13882.79422	13885.07047	4.552494	1.5	0.666666667
30	9.26781	13748.50697	13866.05343	**235.092932**	1.5	6.17854
31	1.68391	13801.41431	13865.4511	**128.073596**	1.5	1.122606667
32	57.98693	13840.05667	13859.40751	**38.701682**	1.5	38.65795333

lnL: log-likelihood scores; n.m: null model; a.m: alternative model; **$p<0.01$, $\chi2= 5.99$.

TableS13. Estimation of adaptive divergence for each branch of the topological distribution of PBV-R_3

Branch No.	ω_2	$-lnL^{n.m}$	$-lnL^{a.m}$	2ΔlnL	kappa	Divergence
1	3.45722	10814.55444	10814.55441	-5.6E-05	5.12	0.675238281
2	1	10794.22518	10814.5544	**40.658448**	5.12	0.1953125
3	1.32185	10772.26816	10814.5544	**84.57248**	5.12	0.258173828
4	70.41316	10751.54355	10813.65484	**124.222594**	5.12	13.75257031
5	71.5113	10814.89513	10814.5544	-0.681464	5.12	13.96705078
6	370.20892	10773.53084	10814.12489	**81.18811**	5.12	72.30642969
7	301.88287	10737.49476	10813.14051	**151.291508**	5.12	58.96149805
8	1	10782.06918	10814.5544	**64.97044**	5.12	0.1953125
9	1.24131	10752.1762	10814.5296	**124.706788**	5.12	0.242443359
10	75.82974	10781.29538	10814.5544	**66.518048**	5.12	14.81049609
11	37.01953	10778.69157	10808.81531	**60.247486**	5.12	7.230376953
12	38.67425	10814.55462	10814.5544	-0.000448	5.12	7.553564453
13	1	10785.77798	10814.5544	**57.55284**	5.12	0.1953125
14	1	10741.49088	10814.01732	**145.052882**	5.12	0.1953125
15	1	10729.00263	10814.5544	**171.10353**	5.12	0.1953125
16	1.42883	10757.43764	10814.5544	**114.23352**	5.12	0.279068359
17	535.57793	10773.07363	10814.43895	**82.730654**	5.12	104.6050645
18	1	10792.89192	10814.5544	**43.324952**	5.12	0.1953125
19	303.56862	10752.21652	10805.83728	**107.24152**	5.12	59.29074609
20	1	10765.0624	10814.5544	**98.983992**	5.12	0.1953125
21	1	10755.87814	10812.2267	**112.697128**	5.12	0.1953125
22	1	10726.55278	10804.93143	**156.757312**	10.38	0.096339114
23	1.41511	10761.26543	10810.7741	**99.017342**	10.38	0.136330443
24	1.90532	10795.25262	10814.5544	**38.603566**	10.38	0.18355684
25	1	10734.47422	10802.66597	**136.383508**	10.38	0.096339114
26	294.92868	10729.00263	10803.15479	**148.30431**	10.38	28.41316763
27	15.67819	10754.04575	10792.95956	**77.827608**	10.38	1.510422929
28	1	10777.11532	10814.5544	**74.878168**	10.38	0.096339114
29	81.76518	10770.20874	10806.64447	**72.871466**	1.69	48.38176331
30	82.98184	10814.55455	10814.5544	-0.000294	1.69	49.10168047

lnL: log-likelihood scores; n.m: null model; a.m: alternative model; $**p<0.01$, $\chi2= 5.99$.

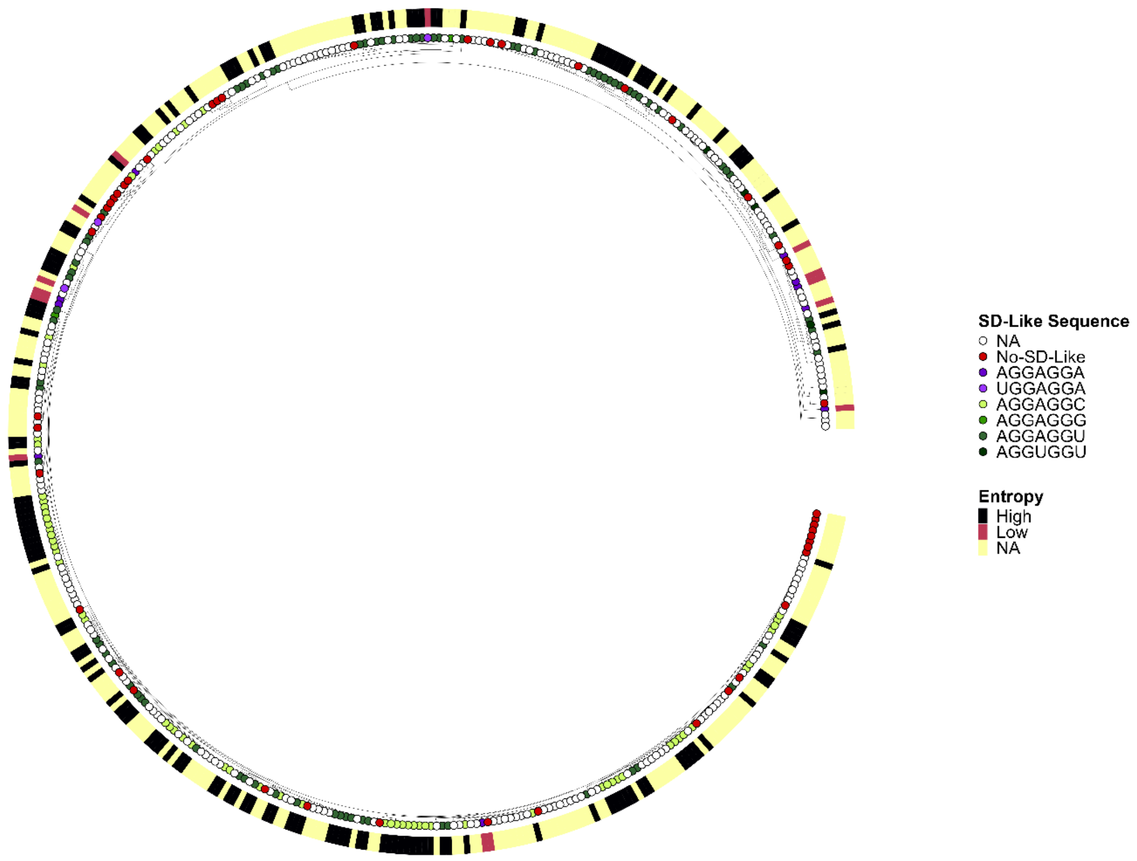

Figure S5. Distribution and variability of Shine-Dalgarno motifs on RdRp segment visualized as integrated phylogeny. Temporal phylogenetic tree based on the coding region of segment 2 (RdRp) reconciled by the SD-Like motif (tips) distribution with stability of the SD-like sequence (determined by entropy of each position

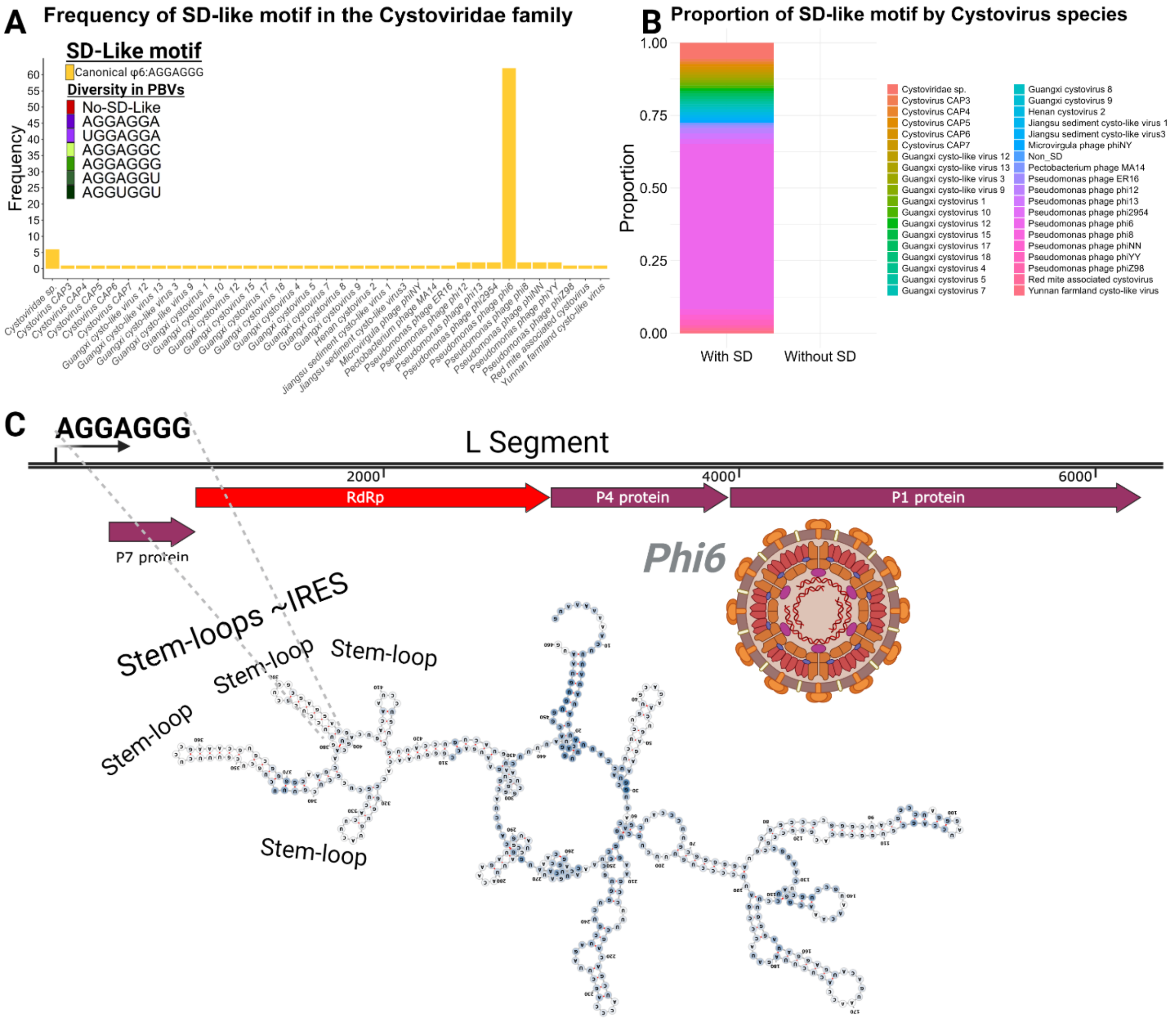

Figure S6. Shine-Dalgarno motifs analysis for all members of *Cystoviridae* family (dsRNA phages) for segment L. A) Evaluation of the potential diversity from the canonical sequence AGGAGGG across the different viral species withing the family. B) Evaluation of the presence of the SD-motif in all the different species of *Cystoviridae* family expressed as proportion of the 109 sequences analyzed (see, Table S14). C) Evaluation of the stability of the the canonical sequence using the 62 sequences available of phi6 where stem-loops were formed, which is recognized as an alternative strategy that resemble the IRES structure of some viruses of bacteriophages as T7.

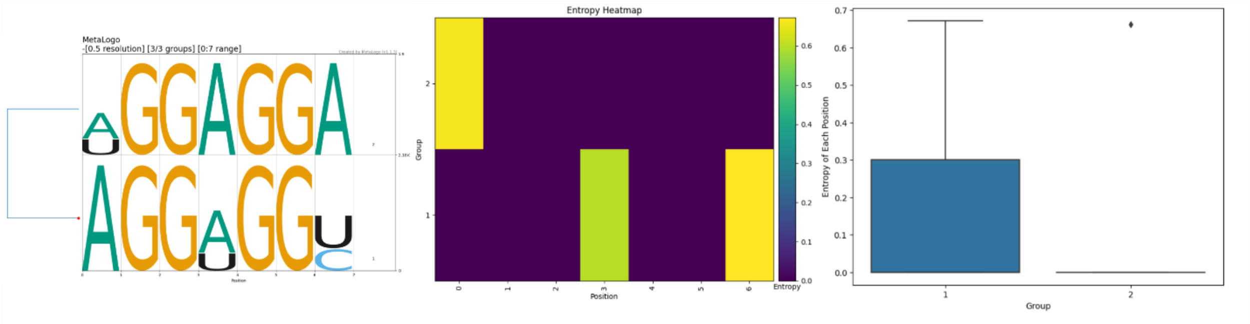

Figure S7. Shine-Delgarno motifs analysis based on stability and group distribution for segment 1 of PBVs. Grouping pattern based on nucleotide composition using the software Metalogo, heatmap of the entropy values for each position, statistical analysis of the entropy value for each group.
